# Role of GABA Receptors in Fetal Lung Development in Rats

**DOI:** 10.1371/journal.pone.0014171

**Published:** 2010-11-30

**Authors:** Narendranath Reddy Chintagari, Nili Jin, Li Gao, Yang Wang, Dong Xi, Lin Liu

**Affiliations:** Lundberg-Kienlen Lung Biology and Toxicology Laboratory, Department of Physiological Sciences, Oklahoma State University, Stillwater, Oklahoma, United States of America; University of Giessen Lung Center, Germany

## Abstract

Fluid accumulation is critical for lung distension and normal development. The multi-subunit γ-amino butyric acid type A receptors (GABA_A_) mainly act by mediating chloride ion (Cl^−^) fluxes. Since fetal lung actively secretes Cl^−^-rich fluid, we investigated the role of GABA_A_ receptors in fetal lung development. The physiological ligand, GABA, and its synthesizing enzyme, glutamic acid decarboxylase, were predominantly localized to saccular epithelium. To examine the effect of activating GABA_A_ receptors in fetal lung development *in vivo*, timed-pregnant rats of day 18 gestation underwent an *in utero* surgery for the administration of GABA_A_ receptor modulators into the fetuses. The fetal lungs were isolated on day 21 of gestation and analyzed for changes in fetal lung development. Fetuses injected with GABA had a significantly higher body weight and lung weight when compared to phosphate-buffered saline (control)-injected fetuses. GABA-injected fetal lungs had a higher number of saccules than the control. GABA increased the number of alveolar epithelial type II cells as indicated by surfactant protein C-positive cells. However, GABA decreased the number of α-smooth muscle actin-positive myofibroblasts, but did not affect the number of Clara cells or alveolar type I cells. GABA-mediated effects were blocked by the GABA_A_ receptor antagonist, bicuculline. GABA also increased cell proliferation and Cl^−^ efflux in fetal distal lung epithelial cells. In conclusion, our results indicate that GABA_A_ receptors accelerate fetal lung development, likely through an enhanced cell proliferation and/or fluid secretion.

## Introduction

The fetal lung undergoes distinct stage-specific changes during gestation. These stages include embryonic, pseudoglandular, canalicular, saccular, and alveolar stages. The duration and developmental changes in each of these stages vary depending on animal species. Particularly interesting are the canalicular and saccular stages where the lungs are progressively filled with increasing amounts of fluid [1]. Lung hypoplasia due to oligohydramnios occurs when fluid secretion is reduced.

The maintenance and secretion of the fluid are required for normal lung development [2]. Distal lung epithelial cells are the sources of this fluid. The fluid is rich in Cl^−^ and low in bicarbonate and proteins. The concentrations of Cl^−^ and bicarbonate (in mM) are 157 and 2.8 in lung fluid when compared to 87 and 19 in plasma. The high concentration of Cl^−^ is due to a high expression of Cl^−^ channels during early pregnancy which are drastically down-regulated at birth [3]. Prior to birth the Cl^−^ secretion is replaced by Na^+^ reabsorption due to various physiological stimuli including epinephrine and thyroid hormones [1]. Up-regulation of Na^+^ channels including Na^+^-K^+^ ATPase occurs at birth to promote fluid reabsorption and facilitate gas exchange [4]. Reduced fluid reabsorption is one of the causes of infant mortality [5].

Fetal lung epithelial cells maintain a very high level of intracellular Cl^−^ ([Cl^−^]_i_) due to basolaterally located Na^+^-K^+^-Cl^−^ cotransporter (NKCC), which mediates Cl^−^ influx utilizing the electrochemical gradient created by Na^+^-K^+^ ATPase. Interestingly, NKCC-1 gene knockout does not result in respiratory defects in mice [6]. On the other hand, Cl^−^ effluxes could be mediated by a number of apical channels including cystic fibrosis transmembrane conductance regulator (CFTR), Ca^2+^-activated Cl^−^ channels, outwardly rectifying Cl^−^ channels, and voltage-gated Cl^−^ channels (CLC). CFTR gene knockout is not lethal in mice [7]. Similarly, lung development is normal at birth in human cases of cystic fibrosis [8]. Knockdown of CLC-2 in fetal lung explants reduces fetal cyst size and transepithelial voltage [9]. However, CLC-2 knockout mice do not have any respiratory anomalies at birth [10]. Although it is possible that gene knockout mice have developed compensatory mechanisms for the loss of genes to prevent mortality, these results raise the possibility that other Cl^−^ channels may contribute to fetal lung fluid secretion.

γ-aminobutyric acid (GABA) receptors are multi-subunit receptors and can be classified into 3 subtypes (A, B and C) based on their pharmacological agonists and subunit composition. GABA_A_ and GABA_C_ are ionotropic whereas GABA_B_ are G-protein-coupled and metabotropic in nature. GABA_C_ receptors mainly consist of ρ1-3 subunits. GABA_A_ receptors are composed of at least 3 main subunits α, β, γ, and accessory subunits such as δ, ε, θ, π, and ρ. Physiologically, GABA receptors are activated upon binding of the ligand, GABA. The synthesis of GABA is catalyzed by different glutamic acid decarboxylase (GAD) isoforms, GAD_65_ and GAD_67_ depending on their affinity to its coenzyme, pyridoxal-5′-phosphate. In neurons, GAD_65_ provides the synaptic GABA whereas GAD_67_ contributes to the cytoplasmic GABA pools. The GAD_65_ knockout mice are susceptible to seizures; however, the GAD_67_ knockout mice die immediately after birth due to respiratory failure [11], [12].

Activation of GABA_A_ receptors leads to opening of the channel and the conductance of Cl^−^ ions. GABA_A_ receptors have been extensively studied in the brain. They are also localized in peripheral tissues. We have reported the expression profiles of GABA_A_ receptor subunits in both adult lung tissue and epithelial cells [13], [14], [15]. Co-immunoprecipitation and biotinylation experiments have shown that γ2, β2, π, α1 and α3 form functional GABA receptors on the apical membranes of adult lung alveolar type II cells. These GABA receptors mediate Cl^−^ effluxes in type II cells. Also, they are critical in maintaining alveolar fluid balance [14]. The genetic deletion of GABA_A_ receptor subunits γ2 and β3 leads to early death [16], [17]. However, some β3 knockout mice survive, develop a cleft palate and show behavioral changes. Genetic deletion of α1, β2, δ, and α6 is not lethal in mice [18], [19], [20]. Neurophysiological changes have been reported due to genetic deletion of GABA_B_ receptor subunits [21].

Our laboratory has reported dynamic expression of GABA receptor subunits during various stages of fetal lung development [22]. Expression of α2, α5, α6, β1, γ3, and ρ3 decreases from day 18 of gestation to adult. The expression of α3, γ1, γ2, θ, and ρ3 is a relatively stable with a slight up-regulation at birth. These subunits may thus have roles in GABA-mediated fetal lung development and during birth. On the other hand, the expression of α1, β2, and π subunits increases from day 18 of gestation to the adult stage, indicating their potential roles in adult type II cells. Interestingly, the knockdown of π subunit in adult type II cells inhibits Cl^−^ fluxes [14]. The expression of α4, ε, and ρ2 subunits is highly variable. In embryonal stages, GABA mediates Cl^−^ effluxes in neuronal and non-neuronal tissues [23]. Since Cl^−^ secretion is crucial for fetal lung development and there are no studies indicating the role of GABA receptors in fetal lung development, we investigated their roles in fetal lung development. We hypothesized that GABA receptors may promote fetal lung development by mediating Cl^−^ efflux. In the long run, modulation of GABA receptors may aid in treating fetal lung developmental anomalies.

## Materials and Methods

### Materials

All the chemicals were purchased from Sigma Aldrich (St. Louis, MO) unless stated. Bicuculline was from MP Biomedicals (Irvine, CA). Rabbit polyclonal anti-GABA, anti GAD 65/67, and rabbit monoclonal anti-Ki-67 and mouse monoclonal anti-α-smooth muscle actin (α-SMA) were from Abcam (Cambridge, MA). Rabbit polyclonal anti-surfactant protein (SP)-C (sc-13979) was from Santa Cruz Biotechnology (Santa Cruz, CA). Rabbit polyclonal anti-clara cell secretory protein (CCSP) was from Seven Hills Affinity Bioreagents (Cincinnati, OH) whereas mouse monoclonal anti-T1α (mucin-type transmembrane glycoprotein expressed on alveolar type I cell) was provided by Dr. Mary C. Williams (Boston University). Avidin-biotic complex (ABC) staining kit for immunohistochemistry was from Vector Laboratories (Burlinghame, CA). Synthetic absorbable and nylon sutures were from Ethicon (Somerville, NJ).

### Animal breeding

Timed-pregnant Sprague-Dawley rats were bred in house. The day that a vaginal plug was found was considered as day 0. At this time, the female rats were housed separately until used for the experiments. The animals were also monitored for weight gain regularly. The rats gained 50–80 grams by day 18 of gestation. The pregnant animals were maintained on a standard breeder diet. All the animal experiments were approved by the Oklahoma State University Animal Care and Use Committee (VM-10–26).

### In-Utero drug delivery

A surgical procedure for *in utero* administration into fetuses of timed-pregnant rats was done as described before [24]. In brief, pregnant rats (day 18 of gestation) were initially anesthetized with 5% isoflurane. The anesthesia was then maintained for the entire procedure with 1.5–2.0% isoflurane. Following induction, the animal was placed in dorsal recumbency and prepared for an aseptic surgery. A lower midline incision was made along the linea alba. The skin was separated from the underlying fascia. The abdominal muscles were then incised to expose the gravid uterus. The uterine horns were gently exposed and the fetuses visualized. Sterile normal saline was used to prevent any drying of the uterus. Fifty microliters of GABA_A_ receptor modulators (5 mM GABA and 2 mM bicuculline) or vehicle (sterile phosphate-buffered saline, pH 7.4 or PBS) were injected into the amniotic fluid of each fetus using a 29-guage needle. Since the administration volume was approximately 10% of the amniotic fluid, the final concentrations of GABA and bicuculline were 500 µM and 200 µM, respectively. These concentrations were similar to our previous *in vivo* studies [14]. The number of fetuses in each pregnant animal varied from 12–14. We injected 3–4 fetuses with each treatment and we had 3–4 treatment groups in one animal. Following the delivery of drugs, uterine horns were gently returned to the abdominal cavity. The abdominal muscle incision was closed by simple interrupted sutures using a 4/0 synthetic absorbable suture material. Later, skin incision was closed by horizontal mattress sutures using 4/0 nylon suture material. The animals were post-operatively administered buprenorphine (0.05 mg/kg b.wt) for 2 days at every 12 hour interval by intramuscular route. The animals were observed daily for wound healing and any signs of infection. Eleven pregnant animals underwent *in utero* surgery for the delivery of GABA_A_ receptor modulators. The number of fetuses injected with each treatment was 19–35.

### Fetal body and lung weights

On day 21 of gestation, the pregnant rats were euthanized by exposing them to 100% CO_2_ and fetuses were immediately isolated from the uterus for further studies. The fetuses were observed for spontaneous movement following their removal from amniotic sacs. The fetuses were gently cleaned to remove amniotic fluid and examined for any gross lesions. We did not find gross lesions. The fetuses were then weighed. We excluded dead fetuses (8/141). Later, fetal lungs were isolated *en bloc* and placed on a blotting paper to remove blood. The left lung from one of the fetuses in each treatment group was immersed in 4% paraformaldehyde for assessing histological changes.

### Lung morphometry

The rat fetal lung sections were stained with hemotoxylin-eosin for monitoring histological changes. Quantification of saccules was done by counting the number of saccules per field in 6–8 randomly chosen fields from each section. The total of 6–8 fetuses from different animals (n = 6–8) were evaluated for lung morphometry. Care was taken not to include large airways and blood vessels in the fields.

### Immunohistochemistry

Immunohistochemistry was performed on 4-µm sections. Lungs from all the treatment groups (in one animal) were mounted in one block for tissue processing. Immunohistochemistry was done exactly as previously stated [25]. Briefly, the slides were first deparaffinized and treated with 3% H_2_O_2_ for 20 minutes. The slides were then subjected for antigen retrieval by boiling in a 20 mM citrate buffer (pH 6.0) for 20 minutes. Later, the tissues were permeabilized with 0.5% Triton X-100 for 10 minutes and blocked by 10% fetal bovine serum for 1 hour. The slides were then incubated with rabbit polyclonal anti-GAD (1∶150), rabbit polyclonal anti-GABA (1∶150), rabbit polyclonal anti-SP-C (1∶100), mouse monoclonal anti-α-SMA (1∶250), rabbit polyclonal anti-CCSP (1∶250), mouse monoclonal anti-T1α (1∶250), and rabbit monoclonal anti-Ki-67 (1∶250) for overnight at 4°C. The slides were washed in 50 mM PBS for 15 minutes and incubated either with goat anti-mouse or goat anti-rabbit antibodies (1∶250) for 1 hour at room temperature. Immunoreactivity was visualized using Vectastain Elite ABC kit. In the negative controls, primary antibodies were omitted. Non-specific rabbit and mouse IgG served as additional negative controls. The total numbers of SP-C-positive cells were counted in three randomly chosen fields for each treatment. Similarly, the numbers of α-SMA-stained small airways and blood vessels were counted in five randomly chosen fields. The number of proliferating cells (in the mesenchyme and epithelium) was quantified by counting Ki-67- positive cells. The imaging was done under low (40×) and high magnification (100×) using Nikon Upright microscope (E600) equipped with SPOT Insight 2 scientific digital camera (Diagnostic Instruments, Sterling Heights, MI).

### Cl^−^ efflux Assay

Cl^−^ efflux studies were done as described before [14]. Fetal distal lung epithelial cells (day 19) were isolated as described previously [22]. In brief, the cells were incubated with the Ringer's solution containing 5 µCi/ml ^36^Cl at 37°C for 30 minutes. After centrifugation, the cell pellets were rapidly re-suspended in 1.2 ml warm Ringer's solution with 100 µM GABA and/or 100 µM picrotoxin, and incubated for 10 minutes. An aliquot of the cells (200 µl) was removed every 2 minutes and quickly centrifuged through 200 µl of silicone oil. The ^36^Cl radioactivity in cell pellets was counted and normalized to milligram protein. The ^36^Cl efflux rate coefficient (k; in min) was determined by linear regression analysis.

## Results

### Immunolocalization of GAD and GABA in fetal lung

**Figure 1:**
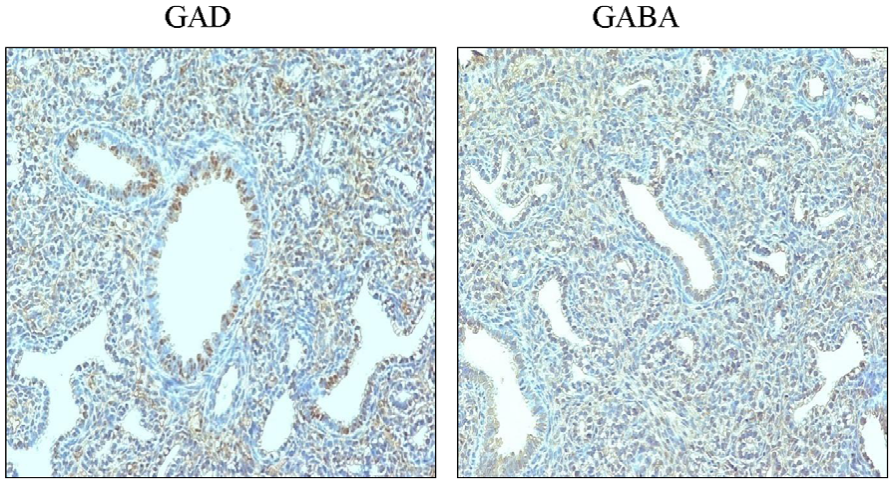
Immunolocalization of GAD and GABA in fetal lungs: Immunohistochemistry was performed to study the immunolocalization of GAD (A) and GABA (B) on fetal lung tissue sections.

The GABA_A_ receptors are activated by the ligand, GABA. We first examined fetal lung tissues for the immunolocalization of the ligand. GABA was localized to a greater extent in the epithelium but to some extent in the mesenchyme (Fig. 1). Thus, our results indicate that the physiological ligand for GABA_A_ receptor activation exists in fetal lungs. We then studied the immunolocalization of the GABA synthesizing enzyme, GAD, in the fetal lungs. GAD was mostly localized to the saccular epithelium (Fig. 1). Thus, fetal lung has machinery for GABA synthesis and the epithelium might act as the physiological source of GABA during fetal lung development.

### Body and lung weights

We delivered GABA *in utero* on day 18 of gestation and analyzed fetal lung development on day 21 of gestation. We did not observe significant mortality following *in utero* surgery. The fetal mortality was 5 percent upon administration of various drugs (5/110). However, bicucuuline when injected alone caused slightly higher mortality (3/31). It is unlikely that bicuculline was toxic because the administration of bicuculline along with GABA did not cause any mortality (0/20). We did not find any significant differences in fetal body weights following the administration of sterile PBS when compared to uninjected fetuses (4.37±0.10 v.s. 4.55±0.11 grams). Fetal body weights were increased by 5% following injection of GABA and bicuclline abolished the increase (Table 1).

**Table 1:** Effect of GABA on fetal body and lung weights during fetal lung development.

Condition	Fetal body weight (% Control)	Fetal Lung weight (% Control)	LW/BW (% of Control)
PBS	100.00±1.28	100.00±2.17	100.00±1.47
GABA	105.34±1.31*	118.26±2.86*	112.36±2.39*
Bicuculline	97.66±1.46	96.66±2.49	99.06±2.20
GABA+Bicuculline	101.43±2.14#	100.92±3.92#	99.15±2.79#

The fetal body weights (BW) in uninjected animals were 4.37±0.10 grams whereas those injected with PBS were 4.55±0.11 grams. Fetal lung weights (LW) in the uninjected and PBS treatment were 131.23±7.05 mg and 136.69±4.36 mg, respectively. The LW/BW (expressed as a percent) in PBS-injected fetuses was 3.02±0.06. Values represent means±SEM from 11–35 fetuses isolated from 4–13 rats. All the values were expressed as percents of PBS control.

*p<0.05 v.s. PBS;

# p<0.05 v.s. GABA.

The administration of PBS did not affect fetal lung weights when compared to uninjected fetuses (131.23±7.05 v.s. 136.69±4.36 mg). GABA significantly increased fetal lung weight (158.76±3.76 mg). The administration of bicuculline did not affect fetal lung weight (129.96±3.98 mg). However, fetal lung weights were significantly reduced following the administration of GABA along with bicuculline (144.83±5.07 mg). Fetal growth was also assessed by wet lung weight-to-fetal body weight ratio (LW/BW) expressed as a percent. A statistically significant increase in LW/BW was observed following administration of GABA (3.38±0.07 v.s. 3.02±0.06). The increase was 12 per cent when compared to PBS-injected fetuses. Bicuculline effectively reduced GABA-mediated increase in LW/BW (3.04±0.09).

### Sacccule number

**Figure 2:**
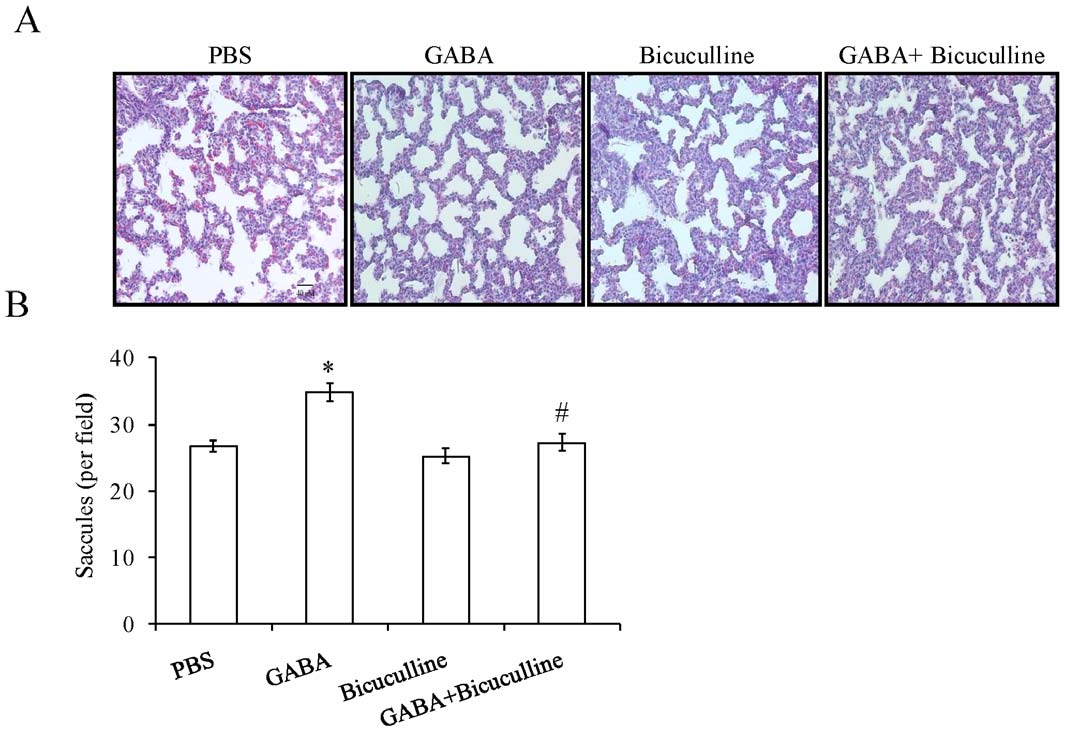
Effect of GABA_A_ receptor modulators on histology and morphometry of fetal lungs: Rat fetuses were injected with PBS, GABA (500 µM), bicuculline (200 µM) or GABA plus bicuculline on day 18 of gestation. The fetal lungs were isolated on day 21. The fetal lung sections were stained with hemotoxylin-eosin (H & E) for studying the histological changes. (A) H & E images (20×) of fetal lungs. Scale bar: 40 µm B) The images were enumerated for number of saccules. Shown are the means±SEM per field. A total of 34–49 microscopic fields from 6–8 animals were evaluated. *p<0.05 v.s. PBS; #p<0.05 v.s. GABA.

Since the administration of PBS did not affect fetal body and lung weights significantly, PBS treatment was used as controls for histological analysis. Histological examination of lung sections clearly indicated the differences in lung structure following *in utero* administration of GABA receptor modulators. GABA reduced thickness of alveolar septa and increased the number of saccules (Fig. 2A). The number of saccules increased to 34.75±1.37 per field following GABA treatment when compared to 26.71±0.89 per field in PBS-injected fetuses (Fig. 2B). Bicuculline alone did not affect the saccule number (25.26±1.14 per field). However, bicuculline decreased the GABA-mediated increase in saccule number (27.29±1.33 per field). Thus, our results indicate that GABA promotes fetal lung maturity.

### Alveolar type II Cells

**Figure 3:**
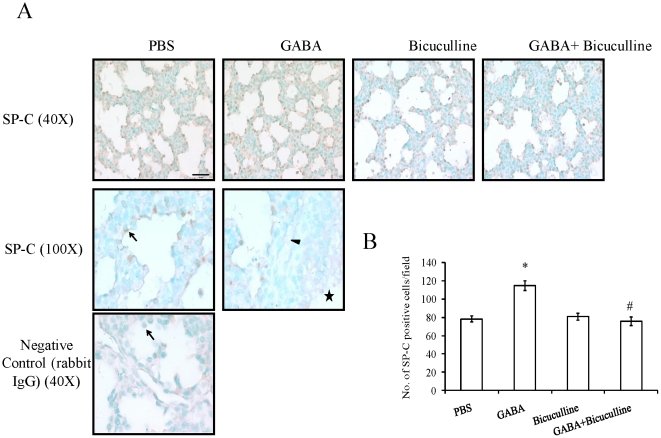
Effect of GABA on SP-C expression during fetal lung development: GABA_A_ receptor modulators, GABA (500 µM), bicuculline (200 µM) or GABA and bicuculline were injected into rat fetuses via *in utero* route of administration. Fetal lungs were isolated on day 21. Immunohistochemistry was done to study the localization of SP-C. (A) Shown are the representative images. Magnified image (100×) indicate specific staining in the saccular epithelial cells (arrow), but not in vasculature (arrowhead) and airway epithelium (asterisk). No specific signals were observed in any of the structures when non-specific rabbit IgG were used. Scale bar: 40 µm. (B) Three random fields per animal were chosen and SP-C-positive cells were counted. Shown are means±SEM of the number of SP-C-positive cells per field. A total of 5 animals were used for the SP-C staining. *p<0.05 v.s. PBS; #p<0.05 v.s. GABA.

We further examined if GABA also affected lung maturity through an increased number of alveolar type II cells. We analyzed the lung sections for the expression of SP-C, a type II cell marker. SP-C protein was localized in epithelial cells, but not in mesenchymal cells (Fig. 3A). Higher magnification images did not reveal staining in airways, blood vessels and mesenchyme. A negative control in which primary antibodies were omitted did not show any signals (data not shown). An additional control using non-specific rabbit IgG also did not have any signals (Fig. 3A). These results indicate the specificity of the antibody used. The numbers of positively stained cells were counted to quantify the changes (Fig. 3B). GABA significantly increased the number of SP-C-positive cells when compared to controls (114.93±6.81 v.s. 78.27±4.41 per field). Bicuculline alone did not affect the number of SP-C-positive cells (80.73±4.82 per field); however, it effectively inhibited GABA-mediated increase in the number of SP-C-positive cells (75.93±6.14 per field). Thus, our results indicate that GABA promotes fetal lung maturity as reflected by an increased number of SP-C-positive cells.

### Myofibroblasts

**Figure 4:**
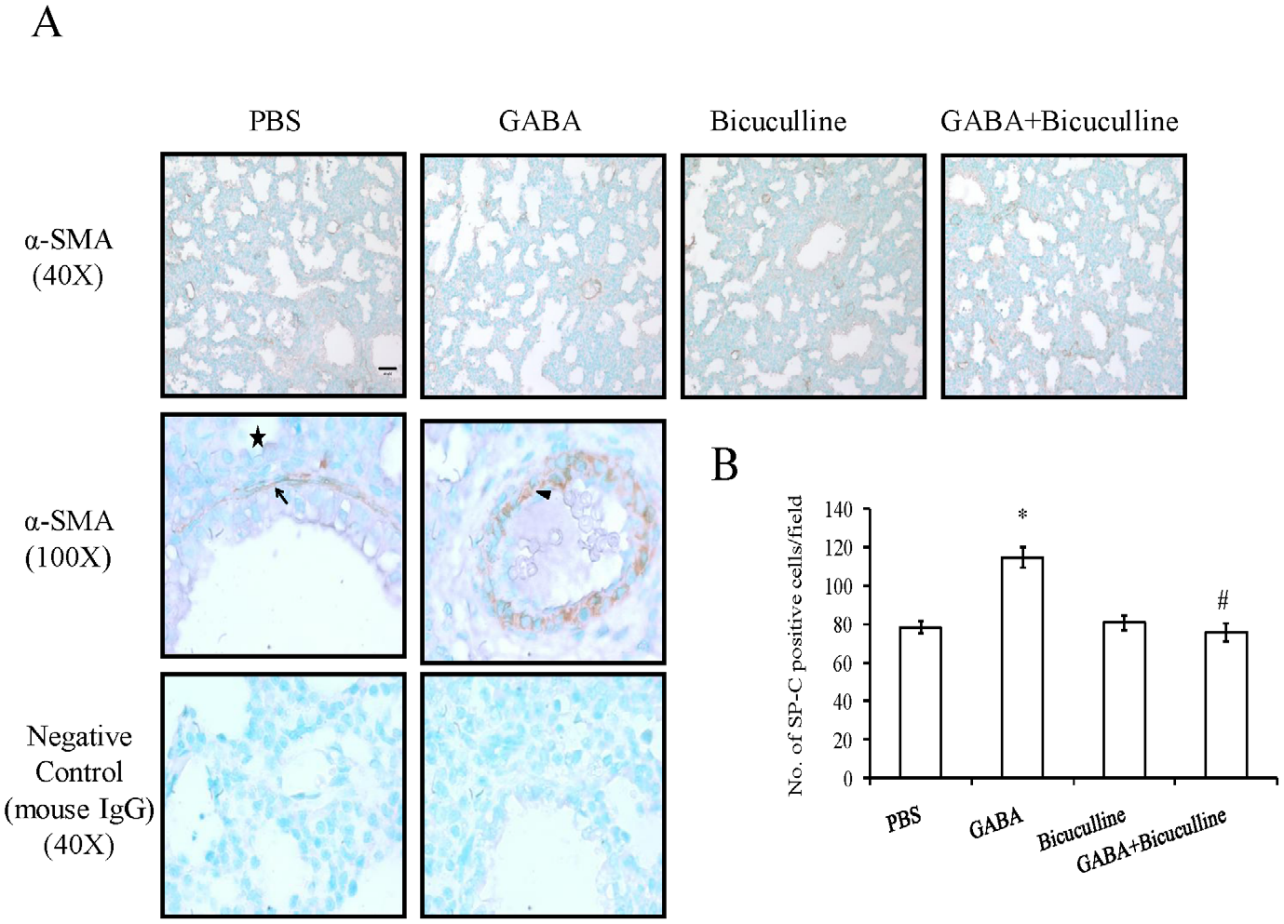
Effect of GABA on α-SMA in fetal lungs: Rat fetuses were injected with GABA receptor modulators. Immunohistochemistry was done for studying the localization of α-SMA, a marker of myofibroblasts. (A) Shown are the representative images. Higher magnification images indicate specific staining of myofibroblasts in airways (arrow) and vasculature (arrowhead), but not in saccular epithelium (asterisk). Negative controls (non-specific mouse IgG) were devoid of any staining. Scale bar: 40 µm. (B) Five random fields per animal were chosen and quantified for α-SMA localization. Shown are means±SEM of the number of α-SMA positive cells per field. A total of 5 animals were used for the α-SMA staining. *p<0.05 v.s. PBS; #p<0.05 v.s. GABA.

Fetal lung undergoes dynamic changes in mesenchyme during development. We monitored the mesenchymal changes by studying the expression of α-SMA following administration of GABA_A_ receptor modulators. α-SMA is expressed by myofibroblast cells in the airway and pulmonary vasculature (Fig. 4A). We did not observe α-SMA expression in saccular epithelium. Negative controls without primary antibodies (data not shown) or with non-specific mouse IgG did not show any signals (Fig. 4A). GABA significantly lowered the number of α-SMA-positive cells in vasculature and small airways when compared to control (7.88±0.54 v.s. 11.88±1.01 per field) (Fig. 4B). Bicuculline alone did not affect the α-SMA expression (12.00±0.68 per field) but effectively inhibited GABA-mediated decreases in α-SMA expression (12.80±0.86 per field). We did not find significant differences in α-SMA staining of the large airways.

### Clara cells and Type I cells

**Figure 5:**
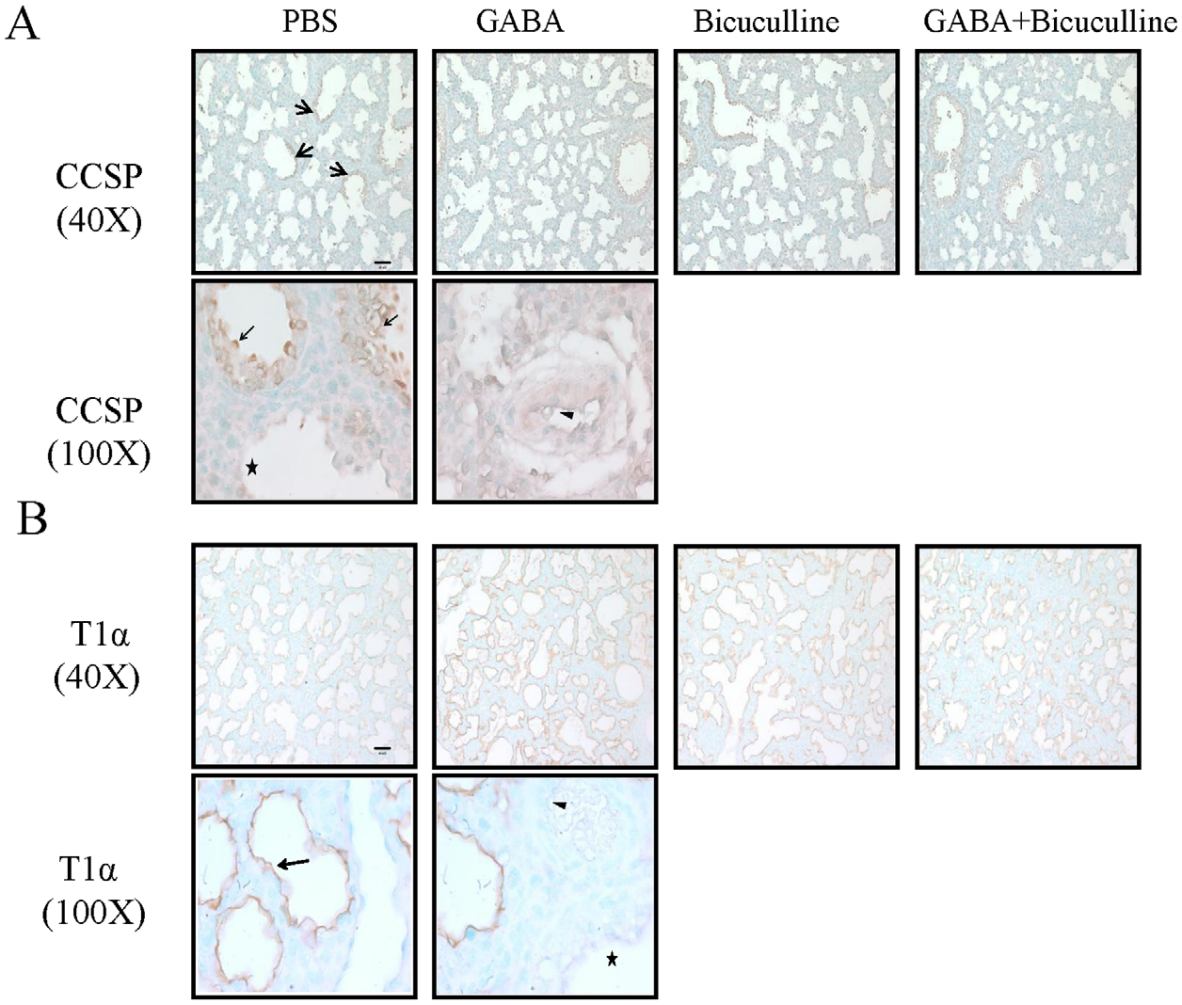
Effect of GABA on CCSP and T1α during fetal lung development: Rat fetuses were injected with GABA (500 µM), bicuculline (200 µM), or GABA plus bicuculline on day 18 of gestation. The fetal lung (day 21 of gestation) tissue sections were processed for studying the localization of CCSP and T1α. (A) The CCSP staining was observed in airways (arrow), but not in saccular epithelium (asterisk) and vasculature (arrowhead). (B) The T1α staining was seen lining the saccular epithelium, but not in airway (asterisk) and vasculature (arrowhead). Shown are the representative images. Scale bar: 40 µm.

We examined the fetal lungs for changes in the expression of CCSP, a clara cell marker localized in upper airways. CCSP staining was observed in airway epithelium but not in saccular epithelium, mesenchyme and blood vessels (Fig. 5A). We did not find significant differences in CCSP staining following GABA treatment. Similarly, the other treatments also did not affect the CCSP staining. Alveolar type I cells can be identified with their marker protein, T1α. Our immunolocalization results indicate that T1α specifically stained the apical membranes of saccular epithelium, but not other structures including airways and vasculature (Fig. 5B). We did not observe any differences in staining pattern of T1α following various treatments. The staining pattern did not allow us to quantify the number of T1α-positive type I cells.

### Cell proliferation

**Figure 6:**
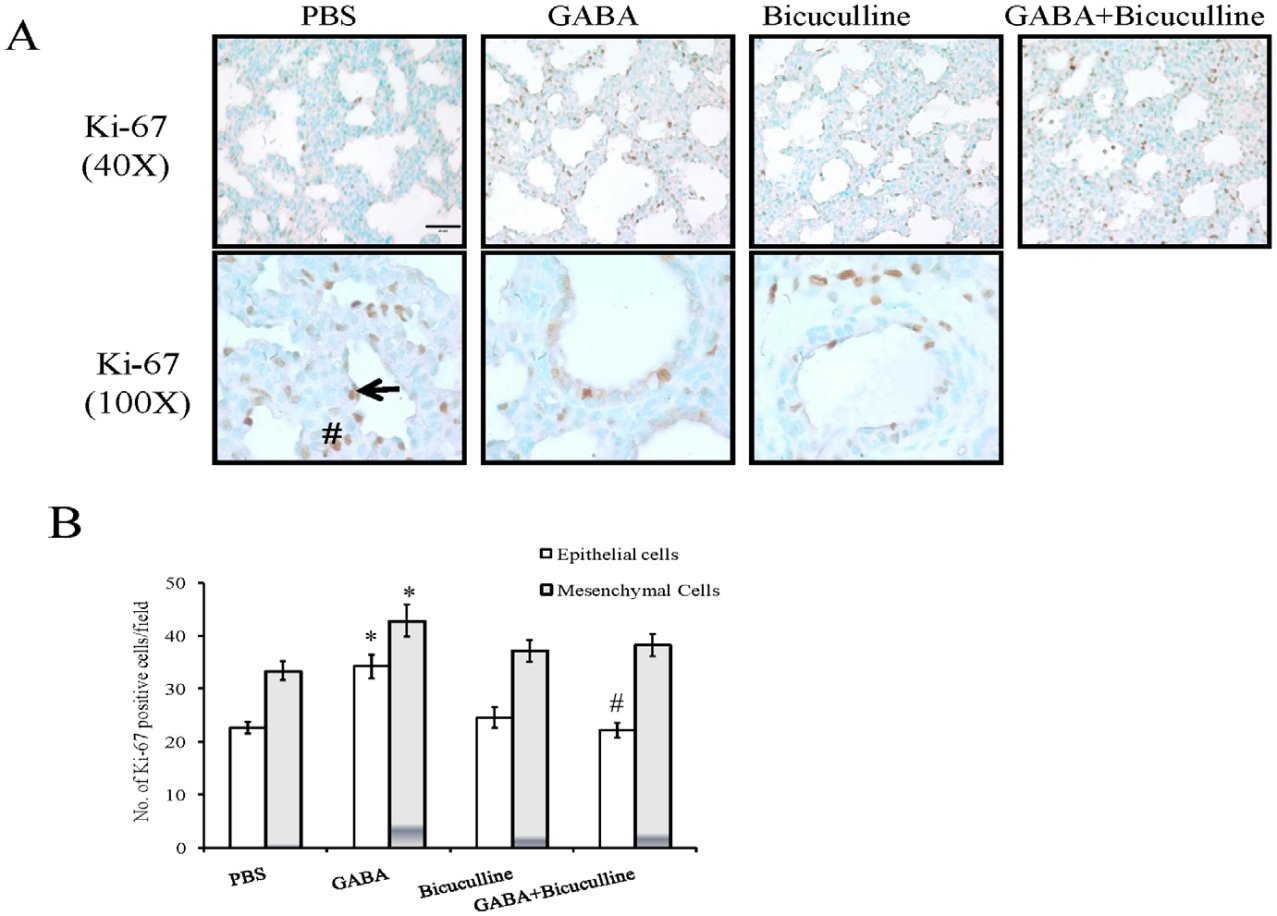
Effect of GABA on cell proliferation during fetal lung development: Pregnant rats at day 18 of gestation underwent *in utero* surgery. The rat fetuses were then injected with PBS, GABA (500 µM), bicuculline (200 µM) or GABA plus bicuculline. Rat fetal lungs were isolated on day 21. Immunohistochemistry was done for the localization of Ki-67, a marker of cell proliferation. (A) Shown are the representative images. Higher magnification revealed specific staining in saccular epithelium (arrow), mesenchyme (pound), airway (asterisk) and vasculature (arrowhead). Scale bar: 40 µm. (B) Three random fields were chosen and quantified for Ki-67 expression in epithelial and mesenchymal cells. Shown are means±SEM (30 microscopic fields) of the number of Ki-67 positive cells per field. A total of 6 animals were used for the Ki-67 staining. *p<0.05 v.s. PBS; #p<0.05 v.s. GABA.

The growth of fetal lung is through the dynamic balance of cell proliferation and differentiation. Cell proliferation was analyzed by enumerating the numbers of Ki-67-positive cells in both the epithelium and mesenchyme. The Ki-67-positive epithelial cells increased significantly following GABA treatment (34.22±2.25) when compared to 26.67±1.06 in PBS- injected fetuses (Fig. 6). Bicucculline alone did not affect proliferation (24.56±1.90), however, it effectively inhibited the GABA-mediated increase in proliferation (22.17±1.44). GABA also increased the number of Ki-67-positive cells (42.89±2.97) in mesenchyme when compared to 33.39±1.80 in PBS injected fetuses. The numbers of Ki-67-positive mesenchymal cells were 37.17±2.11 following the administration of bicuculline alone, and 38.28±2.07 when injected along with GABA. The total number of Ki-67-positive cells in PBS-injected animals was 56.06±2.34 cells. The administration of GABA increased Ki-67-positive cells to 77.11±4.83. Bicuculline did not affect the total number of Ki-67-positive cells (61.72±3.50), however, it significantly decreased GABA-mediated increases in Ki-67-positive cells (60.44±1.95). Thus, GABA promoted epithelial and mesenchymal cell proliferation during fetal lung development.

### Cl^−^ efflux

**Figure 7:**
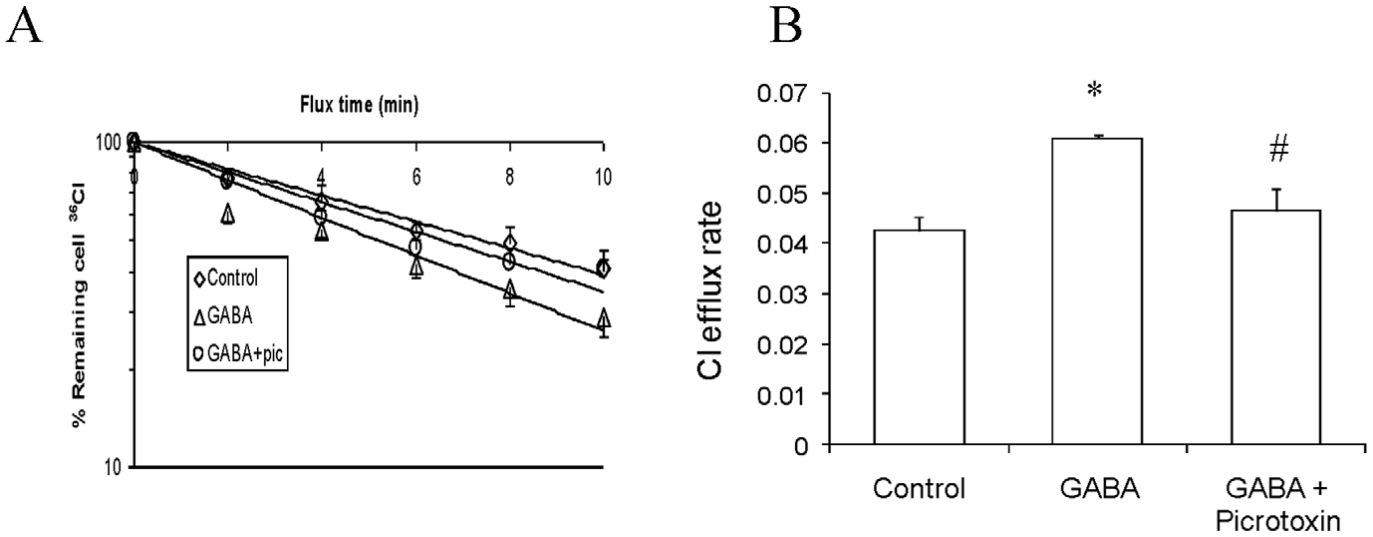
GABA_A_ receptors mediate Cl^−^ efflux in fetal distal lung epithelial cells: Freshly isolated fetal distal lung epithelial cells (day 19) were labeled with ^36^Cl in Ringer's solution. The cells were then incubated without (control) with 100 µM GABA and/or 100 µM picrotoxin (pic), and incubated for 10 min. An aliquot of the cells (200 µl) was removed every 2 min and quickly centrifuged through 200 µl of silicone oil. The ^36^Cl radioactivity in cell pellets was counted and normalized to mg protein. The ^36^Cl efflux rate is expressed as nM/mg/min. (A) Time course, (B) ^36^Cl efflux rate. Shown are means ±SEM. (n = 4 independent preparations). *p<0.05 v.s. control; #p<0.05 v.s. GABA.

GABA_A_ receptor exerts its effects by mediating Cl^−^ fluxes. Our previous study indicated that GABA_A_ receptors mediated Cl^−^ efflux in adult type II cells (15). However, it is unknown if GABA_A_ receptors mediate Cl^−^ fluxes in fetal lung epithelial cells. We hence investigated Cl^−^ fluxes in freshly isolated day 19 fetal distal lung epithelial cells following treatment with GABA alone (100 µM) or along with picrotoxin (100 µM). We observed a net decrease in [Cl^−^]_i_ concentration over a period of time indicating that fetal distal lung epithelial cells actively secrete Cl^−^ into the buffer (Fig. 7A). Cl^−^ efflux (nmol/min/mg protein) in control cells was 0.0627±0.008 (Fig. 7B). GABA increased the Cl^−^ efflux to 0.103±0.013 which was effectively inhibited by picrotoxin (0.0585±0.005).

## Discussion

Fetal lung actively secretes Cl^−^-rich fluid which is critical for lung development. GABA receptors mediate Cl^−^ efflux in embryonal cells and modulate proliferation and differentiation in neuronal and non-neuronal tissues. In the current studies, we found that the GABA synthesizing enzyme, GAD and GABA were localized predominantly to the epithelia of the fetal lung. The *in utero* administration of GABA during fetal stages resulted in higher fetal body and lung weights. GABA increased the number of SP-C-positive type II cells, and decreased α-SMA-positive myofibroblasts. GABA increased cell proliferation and Cl^−^ efflux in fetal distal lung epithelial cells. These results indicate that GABA promotes fetal lung development by increasing proliferation and differentiation of fetal lung epithelial cells.

The physiological concentration of GABA is high in synaptic clefts (in mM); however, the concentration of GABA in fetal lung fluid is unknown. Fetal lungs have the necessary machinery to synthesize and probably secrete GABA into the lung lumen. The released GABA might act in an autocrine and a paracrine fashion to activate GABA receptors in the epithelia and mesenchyme to elicit specific functions.

*In utero* administration (day 18 of gestation) of fluorescent microspheres in mice resulted in their accumulation in lungs and intestines. However, rate of spontaneous abortion was high [26]. We did not observe significant abortion in rats injected with GABA receptor modulators on day 18 of gestation. Previous studies successfully injected adenoviral vectors via *in utero* of administration in rats; however, mostly on 16 day of gestation [24]. The time of administration depends on the aim of the experiment. We have used rats on day 18 of gestation to investigate the role of GABA receptors in the canalicular and saccular stages of lung development.

Intrauterine fetal growth can be measured by fetal birth weight, and fetal organ weights including lungs. A low birth weight is associated with increased risk for respiratory distress syndrome and respiratory diseases in adulthood [27], [28]. *In utero* administration of GABA during fetal stages resulted in a higher body weight, albeit a small increase (5.91±1.22 percent). GABA also promoted fetal lung maturity as indicated by increased lung weights (19.71±2.27 percent). A smaller reduction in fetal body weights (6–8%) and a large reduction in fetal lung weights (25–30%) following pulmonary hypoplasia were also reported in rats [29]. Growth response to tracheal occlusion (TO), a method known to accelerate fetal lung maturity was higher in lungs of rabbits which were more mature at the time of occlusion [30]. The TO at canalicular stage increased LW/BW by 35 per cent. However, TO at the pseudoglandular stage initially increased LW/BW by 15% followed by a robust increase (50%) in the later stages. We instilled GABA_A_ receptor modulators in the canalicular stage of development in rats. It might also be possible that administration of GABA at other stages might alter the outcomes.

GABA-injected fetal lungs had a 30% higher number of saccules when compared to the control. The increased number of saccules indicated that the GABA-treated lungs were more mature. In rats, saccule numbers decreased in nitrofen-induced pulmonary hypoplasia [24]. On the other hand, alveolar saccules increased following tracheal occlusion, a procedure which promotes fluid accumulation and lung maturation [31]. Thus, saccule number can be used as a reliable marker of lung maturity. Increased saccular space with decreased mesenchymal tissue is another indication of fetal lung maturity. We have not fixed the fetal lungs by instillation under constant pressure and hence, the measure of septal thickening might lead to ambiguous conclusions. However, saccule formation is independent of fixation; hence valid conclusions can be made based on saccule quantification [31].

The α-SMA protein is expressed in smooth muscle cells in developing vasculature and airways. α-SMA expression decreased in adult mature lungs when compared to day 21 of gestation and early postnatal life [32]. There was a significantly higher number of α-SMA positive intra-acinar vessels (<30 µm in diameter) in human cases of pulmonary hypoplasia when compared to normal lungs [33]. It is concluded that blood vessel muscularization might be one of etiological factors for vascular changes in fetal lung hypoplasia. In nitrofen-induced pulmonary hypoplasia, mesenchymal tissue was predominant when compared to epithelial cells [34]. GABA decreased the numbers of α-SMA-positive cells. Thus, GABA might have promoted fetal lung maturity by a decrease in number of α-SMA-positive cells.

GABA increased proliferation in Leydig cells [35]. The increased saccule number may be due an increase in type II cell number because GABA increased the number of SP-C-positive cells. Lung expansion promotes growth before and after birth [2]. One of the possible causes of increased lung weights is increased cell proliferation. The increased expression of nuclear antigen protein, Ki-67 is an indication of proliferation since it is not expressed in resting cells. The Ki-67-positive cells were significantly increased following GABA treatment, but the increase was higher in epithelial cells when compared to mesenchymal cells. A higher GABA-mediated proliferation in epithelia might be due to the predominance of GABA receptor subunits such as α4, β1, γ, and π in the epithelium. However, some subunits such as α4 subunits are also present in the mesenchyme [22]. GABA receptors mediate Cl^−^ efflux, leading to opening of volgate-gated calcium channels. The increased intracellular Ca^2+^ concentration may modulate proliferation, migration, and differentiation. The net effect depends on the cell types [36], [37]. TO increased proliferation activity in the epithelium when compared to control fetuses [30].

During fetal lung development, the distal lung epithelial cells continuously secrete Cl^−^ ions which in turn drive the movement of water into the lung lumen. The increased fluid promotes lung development. GABA increased Cl^−^ efflux in adult lung type II cells [14]. Our current study also showed that in fetal distal lung epithelial cells GABA increased Cl^−^ efflux which was inhibited by picrotoxin. Our results are in conformity with previous studies which indicated GABA-mediated Cl^−^ efflux in embryonal cells [23]. Therefore, GABA-mediated increase in epithelial proliferation and differentiation of type II cells might be due to increased Cl^−^ secretion.

Gene knockout studies indicate that genetic loss of some GABA subunits results in severe changes in phenotype. Since GABA is an inhibitory neurotransmitter, the lethality was thought to be due to neuronal changes [17], [20], [21], [38]. Since GABA increases Cl^−^ efflux in fetal distal epithelial cells and promotes fetal lung maturity, pulmonary immaturity observed in knockouts might also be one of the reasons for the lethal phenotype.

Our present study might have significant implication in pulmonary diseases associated with fetal lung development, particularly, those due to impaired fluid secretion. Normal fluid secretion is essential for fetal lung development and maturation. Decreased fluid secretion in oliogohydramnios and congenital diaphragmatic hernia (CDH) leads to pulmonary hypoplasia [39]. Delayed epithelial cell differentiation, along with surfactant protein deficiency, is found in infants with CDH [40]. The CDH is a development anomaly of newborns (1 in 2500 births). CDH accounts for 8% of the major congenital defects and causes significant mortality (50–60%) in United States and developing world [41]. Significant morbidity is noted in surviving infants. The CDH is caused by the rupture of the diaphragm. Herniation of abdominal organs into the thoracic cavity compresses the fetal lung and inhibits its development. Thus, CDH is characterized by pulmonary hypoplasia, left ventricular hypolasia, and pulmonary hypertension [42], [43]. Decreased vasculature with increased musculature predisposes newborns to pulmonary hypertension [44], [45]. Histologically, the CDH lungs have reduced numbers of airway divisions, alveoli and arterial branches.

TO by fetal surgery ameliorates pulmonary hypoplasia due to CDH. The TO increases fluid retention in the lung and thus promotes fetal development in various animal models. However, TO decreases the number of type II cells. Since alveolar type II cells secrete lung surfactant, a reduction in type II cell numbers might reduce lung surfactant in alveolar space and predispose the infants to neonatal acute respiratory distress syndrome [46], [47]. TO procedure in humans did not improve the CDH prognosis [47]. However, an improved method using fetoscopic tracheal balloon occlusion method is under active clinical trials. Our results revealed that GABA increased SP-C-positive type II cells and fetal lung development, opening avenues for use in treating CDH. Even though we used an invasive approach for the delivery of GABA to fetal lungs, it is possible that GABA can be administered parenterally/orally to elicit similar effects.
